# Combined Single-Session Stereotactic Biopsy and Microwave Ablation of Primary and Secondary Liver Tumors

**DOI:** 10.3390/biomedicines13122865

**Published:** 2025-11-24

**Authors:** Liang Zhang, Anthony Ngu, Laura Sophia Kupke, Vinzenz Mayr, Quirin Strotzer, Moritz Brandenstein, Christian Stroszczynski, Ingo Einspieler

**Affiliations:** Department of Radiology, University Medical Center Regensburg, Franz-Josef-Strauss-Allee 11, 93053 Regensburg, Germanyvinzenz.mayr@ukr.de (V.M.);

**Keywords:** liver tumor, hepatocellular carcinoma, liver metastases, microwave ablation, tumor ablation, CT-guided ablation, stereotactic navigation, liver biopsy, single-session

## Abstract

**Objective:** To evaluate the safety, diagnostic yield, and ablation efficacy of a single-session workflow combining stereotactic percutaneous core-needle biopsy (CNB) immediately followed by microwave ablation (MWA) for liver tumors. **Methods:** We retrospectively reviewed consecutive patients (December 2021–May 2025) who underwent stereotactic CNB followed by MWA in the same procedure. Primary endpoints were primary technique efficacy (PTE) and complications. Secondary endpoints were 6-month local tumor progression (LTP) and diagnostic yield. Six-month LTP was summarized using a Kaplan–Meier (KM) point estimate with Greenwood 95% CIs. **Results:** Thirty-three patients underwent single-session biopsy and ablation (33 biopsied; 41 lesions ablated). PTE was 95.1% (39/41); two residual tumors were successfully re-ablated. Six-month LTP was 3.6% (patient level; KM 95% CI 0.0–10.5%) and 2.8% (lesion level; KM 95% CI 0.0–8.2%). There was one major complication (3%, post-ablation abscess) and no minor complications. Adequate tissue was obtained in all biopsies; a definitive diagnosis was established in 88% (29/33): malignancy in 73% (24/33) and benignity in 15% (5/33); 12% (4/33) were nondiagnostic. In the hepatocellular carcinoma (HCC)-suspected subgroup (LI-RADS LR-3 to LR-5; n = 24), all LR-5 lesions were HCC (11/11). Among LR-4 lesions (n = 7), histology showed HCC in 1/7 (14%) and cholangiocarcinoma in 2/7 (29%); 4/7 (57%) were benign or nondiagnostic. Among LR-3 lesions (n = 6), 2/6 (33%) were HCC and 4/6 (67%) were benign or nondiagnostic. In the metastasis-suspected subgroup (n = 9), malignancy was confirmed in 8/9 (89%); 1/9 (11%) was nondiagnostic. **Conclusions:** Single-session stereotactic CNB followed by MWA is feasible and safe, yields diagnostically useful tissue, and achieves high ablation efficacy.

## 1. Introduction

Image-guided thermal ablation is a well-established treatment option for primary and secondary liver tumors [1,2,3,4,5]. Among thermal ablation modalities, microwave ablation (MWA) is increasingly favored for its capacity to achieve rapid and consistent coagulative necrosis through higher energy delivery and deeper tissue penetration, while maintaining effectiveness in proximity to blood vessels due to its reduced sensitivity to perfusion-mediated heat loss [6,7,8].

However, biopsy is often required before ablation. Traditionally, biopsy and ablation are performed as separate interventions. This staged approach can prolong time to treatment, increases patient burden, and requires repeated anesthesia exposures and hospital resources. A combined single-session biopsy and MWA workflow may offer a more efficient alternative by reducing delays and procedural redundancy.

When histological confirmation is indicated, biopsy before ablation is pivotal, enabling immunohistochemistry and panel-based sequencing of actionable and prognostic markers that guide precision care in HCC and metastatic liver disease; therefore, the choice of biopsy technique is critical [9,10,11,12,13,14]. Both FNA and percutaneous CNB are feasible, but CNB is generally preferred for its intact cores, higher diagnostic yield, and suitability for histology, immunohistochemistry, and molecular profiling [15,16,17,18].

Recent advances in stereotactic navigation provide a robust technical basis for single-session biopsy and MWA workflows. These platforms combine multimodal imaging data with three-dimensional segmentation of tumors and risk structures, apply respiratory motion modeling, and enable millimetric instrument tracking with intraprocedural verification. Together, these capabilities allow highly precise coaxial CNB and reproducible antenna placement within a single coordinated plan [19,20,21,22].

However, whether performing biopsy immediately before MWA maintains safety and technical efficacy in the liver remains uncertain. Needle passage may cause bleeding or introduce air, subtly altering lesion morphology and complicating antenna placement; such effects could reduce targeting accuracy, impair uniform energy delivery, and compromise complete coverage. Immediate ablation also precludes repeat sampling if initial tissue is inadequate. Evidence specifically addressing stereotactic, single-session biopsy followed by MWA in the liver is limited: Perrodin et al. reported stereotactic MWA for malignant liver lesions without focusing on hepatocellular carcinoma (HCC) or colorectal liver metastases, and Wu et al. described a small, non-stereotactic, multi-organ coaxial biopsy-MWA series that included six liver lesions [23,24].

To address this gap, we hypothesized that single-session stereotactic CNB followed by MWA would be feasible and safe, achieve high PTE, and provide tissue adequate for histopathology and molecular testing. Our primary aims were to evaluate PTE and complications; secondary aims were to quantify diagnostic yield, explore correlations between histopathologic findings and pre-ablation imaging features, and describe early (6-month) local tumor progression.

## 2. Materials and Methods

### 2.1. Patients and Study Design

This retrospective single-center study included all consecutive patients who underwent single-session stereotactic percutaneous CNB immediately followed by MWA at our institution between 1 December 2021, and 31 May 2025. Eligible patients had either (1) liver lesions suspicious for HCC in the setting of liver fibrosis or cirrhosis, or (2) liver lesions consistent with metastases from a known extrahepatic primary tumor. In all cases, the treatment indication and the need for biopsy were confirmed by a multidisciplinary tumor board. Patients were excluded for any of the following: uncorrectable coagulopathy (e.g., INR > 1.5 or platelets < 50 × 10^9^/L); active systemic infection or cholangitis; lesions abutting hollow viscus organs or gallbladder and lesions immediately adjacent to central bile ducts; inability to tolerate general anesthesia; refractory ascites precluding safe access/ventilation; pregnancy; prohibitive hepatic decompensation.

Written informed consent was obtained from all participants prior to the procedure. The study was conducted in accordance with the Declaration of Helsinki and was approved by the institutional research ethics committee (approval No. 25-4333-104).

### 2.2. Stereotactic Biopsy and MWA

All procedures were performed under general anesthesia in a dedicated interventional CT suite (Somatom Definition Edge, Siemens Healthineers, Erlangen, Germany) using controlled apnea or high-frequency jet ventilation to minimize respiratory motion. Patients were positioned supine or with slight lateral tilt on a vacuum fixation mattress, and radiopaque skin fiducials were applied for stereotactic registration. A dual-energy contrast-enhanced planning CT (arterial and portal venous phases) was acquired after intravenous administration of 100 mL non-ionic iodinated contrast (Accupaque™ 350; iohexol 350 mg I/mL; GE Healthcare Buchler GmbH & Co. KG, Braunschweig, Germany).

The intervention comprised two sequential stereotactically guided phases within the same session, performed with a stereotactic navigation system (CAS-One IR, CAScination AG, Bern, Switzerland): CNB followed by MWA. In patients with multiple hepatic lesions, one representative lesion was biopsied and all visible lesions were ablated during the session.

First, a biopsy trajectory targeting a representative region of the lesion was planned after three-dimensional segmentation. Under stereotactic guidance, a 17-gauge coaxial introducer was advanced along the planned path, and an 18-gauge biopsy device (Mission™ Disposable Core Biopsy Instrument, BD, Franklin Lakes, NJ, USA) was used to obtain at least three cores of 2 cm each. The coaxial system was then withdrawn, hemostasis at the puncture site was confirmed, and specimens were placed in formalin for histopathological analysis.

Second, MWA was performed either via the same skin entry and parenchymal tract as the biopsy or, when needed to optimize ablation coverage, via a separately planned trajectory. We used a non-coaxial technique, advancing the antenna directly rather than through the coaxial biopsy introducer, to avoid upsizing to a larger-diameter coaxial sheath, thereby limiting bleeding risk, and to eliminate heat conduction along the coaxial sheath that can cause tract or skin burns.

Under stereotactic guidance, a microwave antenna (Dophi™ M150E, Surgnova Healthcare Technologies Co., Ltd., Beijing, China; Emprint™ HP, Medtronic, Minneapolis, MN, USA; or NEUWAVE™ PRXT, NeuWave Medical, Inc., Madison, WI, USA) was advanced to the target, and its position was verified on unenhanced CT with adjustments as required. Device selection reflected availability and operator preference, while ablation planning followed a standardized protocol comprising stereotactic CT-guided needle placement, manufacturer-recommended power and duration tailored to lesion size and prespecified minimal ablative margins. Energy delivery (ablation power and duration) followed size-based, system-specific reference data available in the stereotactic navigation system, targeting minimal ablative margins of ≥5 mm for suspected HCC and ≥10 mm for suspected metastases. Other multiphase CT/MRI characteristics (arterial enhancement pattern, portal-venous washout, hepatobiliary-phase signal, LI-RADS category) did not influence energy settings. During stepwise antenna withdrawal, tract ablation was performed to coagulate the parenchymal tract. An immediate postablation contrast-enhanced CT, performed in the same manner as the planning CT, was obtained to confirm complete ablation and exclude immediate complications. On the immediate post-interventional contrast-enhanced CT, lesion coverage and the ablative margin were assessed qualitatively using the AblaSure module integrated in the CAS-One IR stereotactic navigation system (CAScination AG, Bern, Switzerland) to verify circumferential ablation coverage of the target lesions and to exclude obvious residual viable tumor. Margins were not quantified (no millimetric or volumetric measurements). When qualitative assessment suggested insufficient coverage, additional overlapping ablation was performed during the same session at the operator’s discretion.

**Figure 1:**
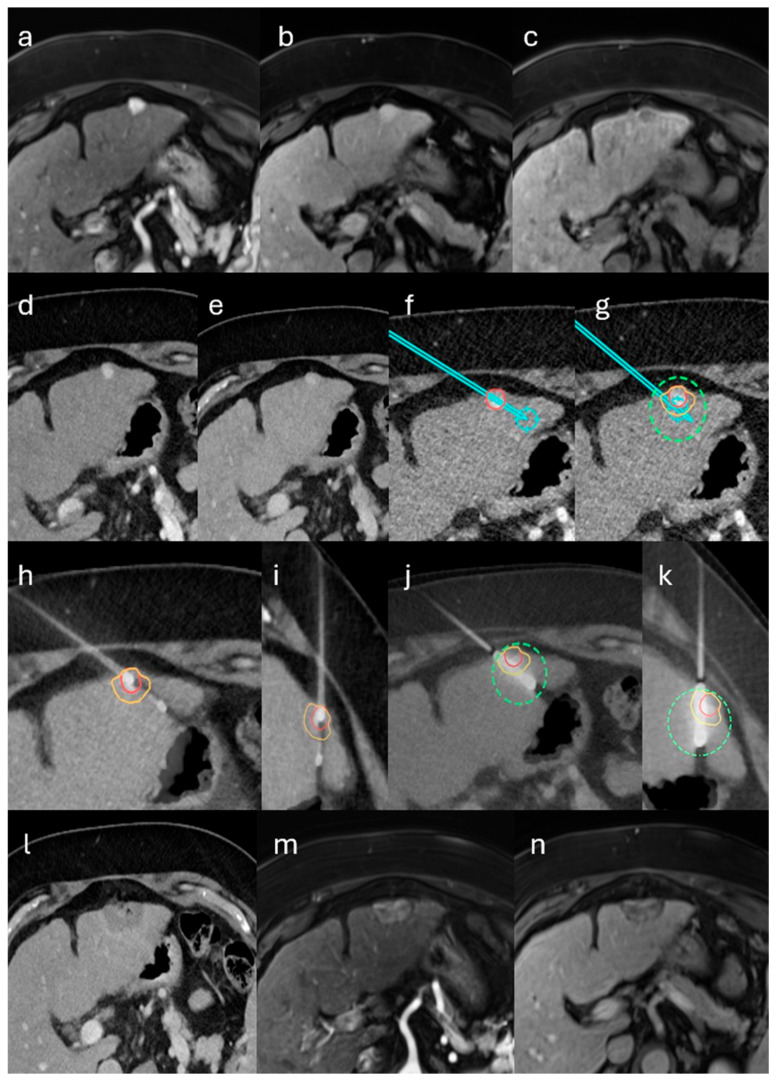
Single-session stereotactic biopsy and microwave ablation (MWA) of an LI-RADS 4 lesion in the left hepatic lobe in a patient with alcoholic cirrhosis. (**a**–**c**) Gadoxetate-enhanced MRI shows a 10-mm subcapsular lesion with non-rim arterial-phase hyperenhancement, no nonperipheral washout on the portal-venous phase, and hepatobiliary-phase hypointensity. Per LI-RADS v2018, major features yield LR-3; the hepatobiliary-phase hypointensity (ancillary feature favoring malignancy) upgrades the category to LR-4. (**d**–**g**) Contrast-enhanced CT–based stereotactic planning with planned coaxial core-needle biopsy and MWA antenna trajectories and predicted ablation coverage including a 5-mm margin. (**h**–**k**) Fused intraprocedural control CT confirms biopsy needle and antenna placement with expected coverage. (**l**) Immediate post-ablation CT demonstrates complete ablation; (**m**,**n**) 6-week MRI confirms sustained complete ablation. Histopathology demonstrated intrahepatic cholangiocarcinoma (CCA). Overlays denote the target lesion (red), planned biopsy and antenna trajectories (cyan path), the predicted ablation zone (green dashed outline), and the intended 5-mm safety margin (yellow or orange).

Per protocol, biopsy and ablation were performed consecutively during a single, uninterrupted anesthesia session. Definitive histology was available only after the procedure; intra-procedural pathology was not obtained, and the immediate ablation decision was not contingent on the biopsy result. Patients with predefined high-risk features were considered at increased risk of post-ablation abscess and, per protocol, would receive prophylactic antibiotics. High-risk features warranting prophylactic antibiotics were predefined as follows: bilioenteric anastomosis or recent biliary instrumentation (such as endoscopic retrograde cholangiopancreatography with sphincterotomy, biliary stent or drain, or pneumobilia). No patient in this cohort met high-risk criteria; therefore, prophylaxis was not administered. Antibiotics were initiated therapeutically only when post-procedural assessment suggested infection, such as compatible clinical signs with elevated C-reactive protein and/or leukocytosis. A representative case of the single-session workflow is illustrated in Figure 1.

### 2.3. Endpoints and Statistical Analysis

The primary endpoints were primary technique efficacy (PTE) and complications. PTE was evaluated on the first follow-up imaging, performed approximately six weeks posttreatment, and defined as complete tumor ablation. Follow-up imaging was primarily performed with liver MRI using a hepatocyte-specific contrast agent (gadoxetate disodium, Primovist^®^, Bayer AG, Leverkusen, Germany); if MRI was contraindicated, multiphase contrast-enhanced CT was used. Follow-up examinations were initially interpreted in routine clinical workflow by abdominal radiologists at our institution. All follow-up examinations were independently reassessed, blinded to procedure details and outcomes, by two abdominal radiologists (4 and 10 years of experience in abdominal radiology). Any discrepancy between readers or with the original report was resolved by consensus. Complications were recorded and graded according to the Cardiovascular and Interventional Radiological Society of Europe (CIRSE) classification system [25].

Secondary endpoints included early local tumor progression (LTP) and diagnostic yield. Early LTP was defined as the appearance of new tumor tissue at or near the edge of the ablation zone within 6 months after an initially successful treatment and was assessed on all subsequent follow-up imaging. Early LTP was summarized at a prespecified 6-month landmark using the Kaplan–Meier point estimate with Greenwood 95% confidence intervals; observations with <6 months of imaging were right-censored at last evaluable scan. Diagnostic yield was defined as the proportion of biopsies that produced a definitive histopathological diagnosis (benign or malignant) among all biopsies performed. Biopsy outcomes were categorized as diagnostic (definitive benign or malignant histopathology) or nondiagnostic (no lesion-specific pathology accounting for the imaging-detected lesion). Nondiagnostic biopsies were further classified according to Kimura et al. as technical error when the biopsy needle did not hit the target tumor (non-lesional hepatic parenchyma on histology or a missed path on imaging) and targeting error when the needle entered the lesion but the sample was insufficient or non-representative for diagnosis (e.g., necrosis, hemorrhage, fibrosis, or scant tumor cells) [26]. For error classification, histology took precedence over imaging.

For diagnostic outcomes, results were analyzed for the overall cohort and by indication (HCC-suspected and metastasis-suspected). Within the HCC-suspected group, lesions were stratified using the Liver Imaging Reporting and Data System (LI-RADS) v2018 into LI-RADS categories LR-3, LR-4, and LR-5 [27].

Continuous variables were reported as median (IQR) or mean ± SD, and categorical variables as counts and percentages. Data normality was tested using the Shapiro-Wilk test. Diagnostic yield was evaluated exploratorily: lesion size was compared between diagnostic and nondiagnostic biopsies using the exact Wilcoxon rank-sum (Mann–Whitney) test, two-sided, and categorical strata were assessed using Fisher’s exact test for subcapsular location, cirrhosis status, and LI-RADS category (within LI-RADS 3–5). A *p*-value < 0.05 was considered statistically significant. Analyses were performed using SPSS Statistics version 28.0 (IBM Corp., Armonk, NY, USA).

## 3. Results

### 3.1. Patient, Tumor and Treatment Characteristics

**Figure 2:**
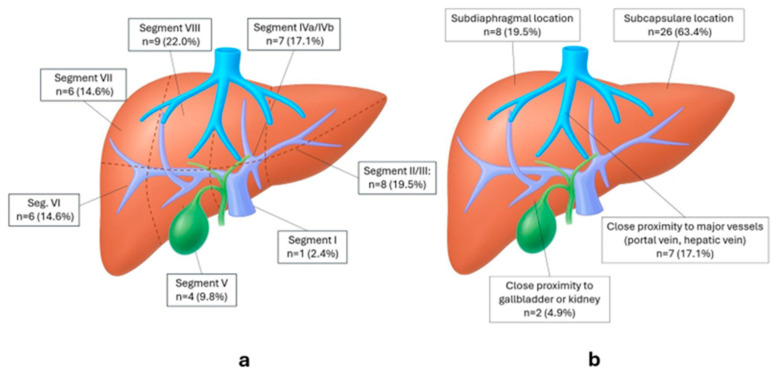
Lesion distribution and location features of all ablated lesions (n = 41). (**a**) Couinaud segment distribution of lesions. (**b**) Location features; categories are not mutually exclusive.

Between December 2021 and May 2025, 33 consecutive patients underwent single-session stereotactic biopsy with immediate MWA (one index lesion biopsied per patient; 41 lesions ablated). Baseline demographics and procedural metrics are summarized in Table 1; segmental and location distributions for ablated lesions are shown in Figure 2.

### 3.2. Primary Technique Efficacy (PTE)

PTE was achieved in 39 of 41 treated tumors (95.1%). By indication, PTE was 25/26 (96.2%) for HCC and 14/15 (93.3%) for metastases; one non-PTE occurred in each subgroup. Per-device PTE was 22/23 (95.7%) for Dophi™, 14/15 (93.3%) for NEUWAVE™, and 3/3 (100%) for Emprint™. Two tumors in two separate patients showed residual viable tissue on first follow-up imaging and were successfully re-ablated in a second session. On retrospective root-cause analysis, the primary driver for non-PTE was motion-related stereotactic misregistration in subdiaphragmatic lesions with low intraprocedural conspicuity.

### 3.3. 6-Month Local Tumor Progression (LTP)

One LTP event was detected 107 days after the procedure. At the patient level (n = 33), the 6-month LTP by Kaplan–Meier was 3.6% (95% CI 0.0–10.5%), with 22 patients still under surveillance at 6 months, 10 censored before 6 months, and 1 event before 6 months. At the lesion level (41 lesions), the 6-month LTP was 2.8% (95% CI 0.0–8.2%), 14 lesions were censored before 6 months and 26 remained at risk.

### 3.4. Complications

Among 33 procedures, a single complication (3%) occurred: one patient developed a liver abscess one week after ablation, which was successfully treated with percutaneous drainage and antibiotics. No other complications were observed. This event was classified as a major complication (CIRSE grade 3) due to the need for additional therapy and hospitalization.

### 3.5. Histopathological Yield and Correlation with Imaging

**Figure 3:**
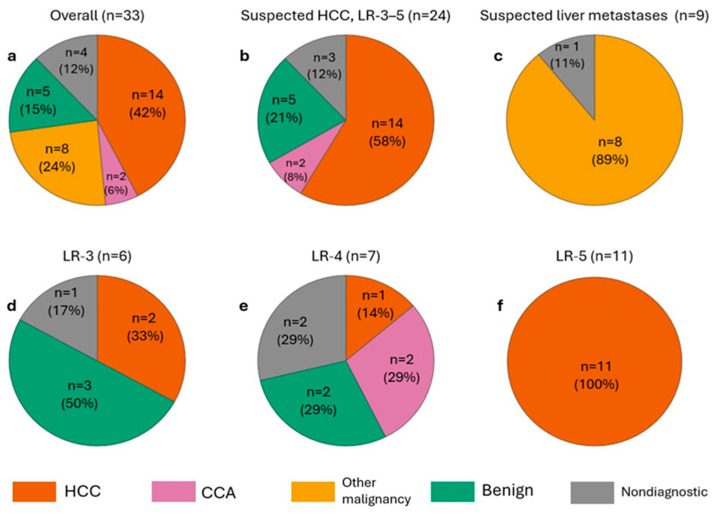
Distribution of biopsy diagnoses across cohorts and LI-RADS categories. (**a**) Overall study population. (**b**) Suspected HCC (LR 3–5). (**c**) Suspected liver metastases (known primary). (**d**–**f**) Subgroups of (**b**): LR-3, LR-4, LR-5. The benign lesions shown were ablated as part of the single-session protocol; definitive histology was available only post-procedure and therefore did not influence the immediate treatment decision. Percentages are rounded to whole numbers and may therefore not add up to exactly 100%. Abbreviations: HCC, hepatocellular carcinoma; CCA, cholangiocarcinoma; LR, LI-RADS category.

Adequate tissue for histopathological analysis was obtained in all cases. Figure 3 illustrates the overall and subgroup distributions.

In the LI-RADS 3–5 (HCC-suspected) subgroup (n = 24), all LR-5 lesions were HCC and the two CCA diagnoses occurred among LR-4 lesions. Benign findings comprised two regenerative nodules, one focal nodular hyperplasia, one inflammatory nodule, and one sclerosed hemangioma. In the metastasis-suspected subgroup (n = 9), 8/9 lesions (89%) were malignant and concordant with the known primary tumor, and 1/9 (11%) was nondiagnostic.

Nondiagnostic results occurred in 4/33 biopsies (12%). In every nondiagnostic case, histology showed only non-lesional hepatic parenchyma without lesion-specific pathology to account for the imaging findings. Although intraprocedural control scans suggested needle passage through the intended target, we classified these as technical errors, prioritizing histologic evidence that the target was not sampled; no targeting errors were recorded. Nondiagnostic lesions were smaller (median 10 mm, IQR 6–14) than lesions with a diagnostic outcome (16 mm, IQR 10–21), but the difference did not reach significance (exact Mann–Whitney U, two-sided *p* = 0.108). Diagnostic yield did not differ significantly by subcapsular location (nondiagnostic 0/12 non-subcapsular vs. 4/21 subcapsular; Fisher’s exact *p* = 0.271), cirrhosis (3/14 without vs. 1/19 with; *p* = 0.288), or LI-RADS category within the LR3–5 cohort (nondiagnostic 0/11 LR-5 vs. 3/13 non-LR-5; *p* = 0.223).

## 4. Discussion

This retrospective study evaluated a single-session, stereotactic workflow that integrates percutaneous liver biopsy with immediate MWA. To our knowledge, it is among the first to systematically assess same-session biopsy and MWA, reporting diagnostic yield, technical efficacy, safety, and imaging–pathology concordance across common indications such as hepatocellular carcinoma and colorectal liver metastases.

PTE was achieved in 95.1% of treated lesions, with two tumors requiring repeat ablation. These results are consistent with pooled stereotactic and robotic thermal ablation PTE (94% at 1–6 weeks; 90% at 6–12 weeks) reported by the meta-analysis of Tinguely et al. [28]. Methodologically comparable stereotactic MWA studies report PTE between 88–97% [19,23,29,30,31,32,33].

The combined biopsy–MWA approach was well tolerated, with only one major complication (3%), a post-ablation liver abscess managed with antibiotics and percutaneous drainage (CIRSE grade 3), and no minor complications across 33 procedures. The abscess occurred in a patient who was not deemed high risk and therefore did not receive prophylactic antibiotics. Our protocol incorporated safeguards, including exclusion of patients with active infection or cholangitis, exclusion of lesions immediately adjacent to central bile ducts, and routine tract ablation during antenna withdrawal. Although a biopsy–then–ablation sequence could theoretically increase contamination risk, this single event in a small cohort is insufficient to infer a higher infection risk linked to the single-session workflow. Within these limitations, the complication profile is consistent with contemporary stereotactic MWA series and aligns with the meta-analysis by Tinguely et al., which reported an overall complication rate of 11.4% and a 3.4% major-complication rate for stereotactic/robotic liver thermal ablations [19,23,28,29,30,32,33,34]. Larger prospective studies are needed to more precisely estimate abscess risk and to assess whether standardized prophylaxis benefits selected subgroups in this single-session biopsy–MWA workflow.

Six-month LTP was 3.6% at the patient level (KM 95% CI, 0.0–10.5%) and 2.8% at the lesion level (KM 95% CI, 0.0–8.2%). Direct comparisons across studies are difficult because follow-up windows, imaging protocols, cohort composition and size differ; nevertheless, we cite two contemporary datasets for context. The NeuWave Observational Liver Ablation (NOLA) registry (615 patients; 760 tumors; mixed primary and metastatic disease) reported a 12-month cumulative LTP incidence of 11.9% at the patient level in routine practice [35]. In a stereotactic MWA cohort, Tinguely et al. (153 patients; 301 lesions; mixed HCC and metastases) reported a 6-month per-lesion LTP of 22% [19]. Despite differences in follow-up, and cohort size and mix that limit strict comparability, our LTP rates compare favorably with these benchmarks.

Adequate tissue for histopathological analysis was obtained in all cases, yielding a definitive diagnosis in 88% (29/33). The remaining 12% (4/33) were nondiagnostic, with histology showing only non-lesional hepatic parenchyma, and were categorized as technical errors per Kimura et al. [26]. Although intraprocedural control scans suggested traversal of the radiologic target in each nondiagnostic case, classification followed the histologic findings; therefore, these cases were not categorized as targeting errors. The most plausible driver is the small lesion size in our cohort (median size 14 mm), in which respiratory drift, partial-volume effects, and needle deflection more readily yield non-lesional cores despite apparent overlap on intraprocedural CT. Supporting this size effect, Kimura et al. (n = 938; predominantly ultrasound-guided with a CT-guided subset) reported a 17.2% nondiagnostic rate due to technical error for CT-guided biopsies of liver tumors ≤ 17 mm [26]. Given a median lesion size of 14 mm, our 12% nondiagnostic rate, all classified as technical errors per our predefined criteria, aligns with these findings.

Against an optimized stereotactic benchmark, our diagnostic yield of 88% is lower than the 97.8% reported by Schullian et al. for CT-guided stereotactic liver biopsy; however, their cohort included larger tumors (median 3.5 cm, range 1.5–8.0 cm) and a larger biopsy device (16 G with a 15 G coaxial introducer), whereas our cohort’s median tumor size was 1.4 cm and we used a smaller biopsy device (18 G with a 17 G coaxial introducer) [36]. These differences in lesion size and needle gauge likely contributed to the difference in diagnostic yield. In the broader literature of non-stereotactic CT- or ultrasound-guided liver biopsy, large cohorts report overall diagnostic accuracy spanning ~73–98% [18,37,38,39,40,41]. Within that context, our 88% yield is consistent with contemporary non-stereotactic series.

In our cohort, LR-3 and LR-4 lesions demonstrated a heterogeneous diagnostic spectrum, encompassing benign entities, non-diagnostic results, HCCs and malignant tumors that were not HCC, all of which were intrahepatic cholangiocarcinomas. This variability is consistent with published data showing that the positive predictive value for HCC is substantially lower in LR-3 and LR-4 categories than in LR-5. For example, in the meta-analysis by Lee et al., the pooled proportion of HCC was only 38% for LR-3 and 73% for LR-4, versus 96% for LR-5 [42]. Choi et al. demonstrated that a notable subset of LR-4 lesions ultimately represented intrahepatic cholangiocarcinoma or combined HCC–CCA rather than HCC [43]. These findings underscore the diagnostic uncertainty of indeterminate LI-RADS categories and highlight the utility of same-session biopsy in uncovering clinically relevant alternative pathologies that may critically alter management.

Equally important are the diagnostic advantages: biopsy before ablation secures tissue for histology and molecular testing, ensuring diagnostic certainty and preserving samples for genomic analysis. Biopsy-secured tissue supports precision treatment. In HCC, histology distinguishes HCC from CCA or combined tumors and enables immunohistochemistry and panel-based sequencing to define angiogenic and immune phenotypes and to detect less common, but trial-relevant, genomic alterations [44,45]. Similarly, in metastatic colorectal cancer, biomarker testing for KRAS/NRAS, BRAF V600E, MSI, HER2, and NTRK fusions is essential for selecting systemic therapy and clinical trials [2,46].

A stereotactic single-session biopsy and microwave ablation workflow may offer pragmatic advantages over staged pathways. By consolidating preprocedural assessment, anesthesia, and image-guided interventions into one encounter, it could reduce visits and anesthetic exposures, ease coordination, and potentially accelerate initiation of local therapy. Obtaining tissue in the same sitting may allow histologic confirmation without delaying treatment and can facilitate timely molecular or genomic profiling when indicated. The integrated set-up could also streamline resource use, thereby potentially improving throughput and reducing scheduling-related attrition, particularly among patients who are frail, have limited access to care, or face rapidly evolving disease.

In our series, all procedures were completed in a single session, and we report observed interventional time and anesthesia duration to characterize resource use. However, these inferences are hypothesis-generating: this is a retrospective, consecutive single-arm analysis from a single center with a limited sample, without a control cohort or formal data on time-to-treatment, cost, or patient-reported outcomes. Unmeasured confounding, selection/referral bias, and site-specific logistics may influence apparent efficiencies and limit generalizability. Accordingly, we refrain from comparative claims regarding time-to-treatment, anesthesia exposures, or hospital resource utilization; these endpoints should be tested and quantified in prospective, comparative studies that include time-motion and cost-effectiveness analyses and capture patient-centered outcomes.

This study is limited by its retrospective, single-center design and moderate sample size. Follow-up was heterogeneous and reflects early surveillance only; long-term outcomes were not assessed. With only one LTP by 6 months and substantial early censoring (particularly at the lesion level), confidence intervals are wide and time-to-event estimates imprecise; accordingly, we report a prespecified 6-month LTP and do not estimate a median time to LTP. The absence of a control group (e.g., staged biopsy and ablation) precludes causal inference and prevents quantification of any comparative advantages in time-to-treatment, number of anesthesia exposures, hospital resource use, costs, or oncologic outcomes; the resource-use metrics reported here are descriptive only, and formal cost data were not collected. The mixed HCC/metastasis cohort and the MWA device heterogeneity across three platforms may introduce residual confounding that the study is not powered to resolve. Despite these limitations, the study provides valuable real-world evidence on the feasibility and safety of single-session stereotactic biopsy and MWA, and further multicenter prospective studies are warranted to validate these findings.

## 5. Conclusions

Single-session stereotactic biopsy followed by MWA is a safe, technically effective integrated strategy for liver tumor management. It provides high diagnostic yield with reliable histopathologic confirmation, maintains high ablation efficacy, and has a low complication rate. Further prospective comparative studies are needed to validate these findings and quantify any advantages relative to staged management, in which biopsy and ablation are performed as separate procedures.

## Figures and Tables

**Table 1:** Patient characteristics, indications, tumor features, and interventional metrics.

Characteristics	Value
**Demographics**	
Number of patients	33
Age, years (mean ± SD)	65.1 ± 8.3
Sex—Male	19 (58%)
Sex—Female	14 (42%)
**Liver status**	
Cirrhotic	19 (58%)
— Child-Pugh A	13
— Child-Pugh B	6
Non-cirrhotic	14 (42%)
**Indication**	
Suspected HCC	24 (73%)
— LI-RADS 5	11 (46% of HCC)
— LI-RADS 4	7 (29% of HCC)
— LI-RADS 3	6 (25% of HCC)
Suspected metastatic liver tumors	9 (27%)
— Colorectal cancer	5 (15%)
— Pancreatic cancer	2 (6%)
— Breast cancer	1 (3%)
— Thyroid cancer	1 (3%)
**Tumor counts and size**	
Number of biopsied tumors (1 per case)	33
Number of ablated tumors	41
Tumor diameter, mm (median [IQR])	14 [10–20]
**Tumor size categories**	
— Tumor diameter < 10 mm	16 (39%)
— Tumor diameter 10–20 mm	16 (39%)
— Tumor diameter > 20 mm	9 (22%)
**Interventional metrics**	
Total anesthesia time, minutes (median [IQR])	118 (104–155)
Intervention time, minutes (median [IQR])	79 (60–97)
— Biopsy time, minutes (median [IQR])	10 (8–13)
— Ablation time, minutes (median [IQR])	36 (28–46)
— Other intraprocedural time, minutes (median [IQR])	28 (22–42)
Number of control scans per case (median [IQR])	4 (2–6)
CT DLP per case, mGy·cm (median [IQR])	2110 (1928–2570)
MWA antenna (per ablation)	
— NEUWAVE™ PRXT	15 (37%)
— Dophi™ M150E	23 (56%)
— Emprint™ HP	3 (7%)
Postinterventional hospital stay, days (median [IQR])	2 (2–2)

**Definitions**. Intervention time: from the start of the first intraprocedural planning CT to the end of the final post-ablation control scan. Biopsy time: from biopsy-needle skin puncture to completion of the final tissue acquisition with needle withdrawal; includes targeting/verification scans and needle repositioning. Ablation time: from first ablation antenna skin puncture to final ablation antenna removal; includes verification scans, antenna repositioning, and tract cauterization. Other intraprocedural time: consists of pre-puncture planning/trajectory work-up, post-withdrawal hemostasis, and the final post-ablation control scan.

## Data Availability

The data of this study are available from the corresponding author upon reasonable request.
